# Sexual arousal intensity modulates copulatory behavior and semen quality in Arabian stallions: Effects of age, origin, and collection method

**DOI:** 10.14202/vetworld.2025.2615-2625

**Published:** 2025-09-06

**Authors:** Amel Najjar, Alma Dhaouadi, Sofiane Ezzar, Belgacem Benaoun, Sana Khaldi

**Affiliations:** 1 Laboratory of Animal Genetic and Feed Resources LR15AGR01, National Agronomic Institute of Tunisia, University of Carthage, 1082 Tunis, Tunisia; 2 National Foundation for the Improvement of the Horse Breed, Ministry of Agriculture, Water Resources and Fishes, 2020 Sidi Thabet, Ariana, Tunisia; 3 Laboratory of Epidemiology of Enzootic infections in Domestic Herbivores LR16AGR01, National School of Veterinary Medicine of Sidi Thabet, University of La Manouba, 2020 Sidi Thabet, Ariana, Tunisia

**Keywords:** Arabian stallions, artificial insemination, copulatory behavior, semen quality, sexual arousal

## Abstract

**Background and Aim:**

Stallion sexual behavior during semen collection can be influenced by multiple factors, yet the role of sexual arousal intensity remains underexplored. Understanding how arousal modulates behavioral and physiological reproductive traits is essential for improving artificial insemination (AI) efficiency and semen quality. This study aimed to evaluate the effects of sexual arousal intensity, age, origin, and semen collection method on stallion sexual behavior and the quality of fresh and frozen semen.

**Materials and Methods:**

Thirteen Arabian stallions (7 Tunisian, 6 foreign parentage) aged 6–20 years (total ejaculates = 49) were assessed during semen collection using either a dummy or an estrous mare. Sexual arousal intensity was scored on a four-point scale (− to +++). Behavioral responses, mounting and erection parameters, and semen traits were recorded. Fresh semen was evaluated for volume, motility, concentration, and morphology; frozen semen was assessed for motility, viability, membrane integrity, and abnormalities. Data were analyzed using multifactorial analysis of variance with significance at p < 0.05.

**Results:**

High arousal (+++) increased vocalizations, anogenital sniffing, and Flehmen responses (p < 0.05) but prolonged preparation time (p = 0.05). Low arousal (−/+) prolonged full erection duration (p < 0.01) and improved sperm motility in fresh and frozen semen (p < 0.01). Stallions of foreign origin exhibited higher fresh semen motility (p < 0.01) but required longer preparation and collection times (p < 0.05). The estrous mare method improved fresh semen motility and concentration (p < 0.05) but did not affect frozen semen traits. Younger stallions produced larger semen volumes, but had higher abnormal sperm counts in fresh samples, whereas older stallions showed more abnormalities post-freezing (p < 0.01).

**Conclusion:**

Sexual arousal intensity significantly modulates both behavioral and semen quality parameters in stallions. Lower arousal is associated with prolonged erection and superior sperm motility, suggesting dissociation between behavioral excitement and physiological semen traits. Collection from an estrous mare can enhance fresh semen motility, though the dummy remains safer for handlers. These findings highlight the importance of tailoring semen collection protocols to individual stallion profiles to optimize AI outcomes.

## INTRODUCTION

High-performance stallions are typically introduced into breeding programs at stud farms and equine semen production centers following the completion of their racing careers. Semen is collected on owner’s request and used in artificial insemination (AI) programs to disseminate desirable genetic traits and produce high-performance offspring. However, variability in stallion fertility can limit the overall success of AI programs. Sexual behavior during semen collection, as well as semen quality, can be influenced by several factors, including season [1–3], age [4, 5], prior training with the dummy and artificial vagina [6], and semen handling techniques [7, 8]. These factors collectively contribute to the observed variability in stallion fertility. Evaluating stallion sexual behavior during semen collection is crucial for assessing semen quality, predicting reproductive performance, and optimizing the efficiency of the collection process. Typically, sexual behavior in stallions begins with initial signs of interest toward the dummy or mare, followed by copulatory behaviors such as mounting frequency, erection, and ejaculation timing [9]. Notably, stallions can differ markedly in their behavioral expression; some exhibit pronounced activity and excitement, whereas others remain comparatively passive or indifferent [10].

While numerous studies have examined the influence of environmental, seasonal, and physiological factors on stallion fertility, the role of sexual arousal intensity during semen collection remains poorly characterized. Existing research has largely focused on broad sexual behavior parameters, such as libido scores, mounting frequency, and erection timing, without exploring how varying arousal levels modulate both behavioral responses and semen quality outcomes in fresh and frozen samples. Furthermore, previous work has rarely accounted for the combined effects of stallion origin, age, and semen collection method on these traits. The few studies that have evaluated behavioral factors have often relied on small or homogeneous populations, with limited statistical integration of behavioral and physiological data. Consequently, there is a lack of evidence on whether high or low arousal states consistently enhance or impair semen characteristics, and how such effects interact with genetic background and collection technique. Addressing this gap is critical for refining AI protocols and improving semen collection efficiency, safety, and fertility outcomes in breeding programs.

This study aimed to investigate the influence of sexual arousal intensity on the copulatory behavior and semen quality of Arabian stallions, with a specific focus on identifying how these effects are modulated by stallion age, genetic origin, and semen collection method (dummy vs. estrous mare). The research objectives were: (i) to quantify the relationship between arousal intensity and key behavioral parameters during semen collection, including preparation time, mounting frequency, and erection duration; (ii) to evaluate how arousal intensity affects semen characteristics in both fresh and frozen states, including motility, concentration, morphology, viability, and membrane integrity; and (iii) to determine whether age, origin, and collection method independently or interactively influence these outcomes. By integrating behavioral scoring with objective semen analysis, this study seeks to provide evidence-based recommendations for optimizing stallion management in AI programs.

## MATERIALS AND METHODS

### Ethical approval

The study followed ethical guidelines for the use of animals in research [11] and complied with Tunisian legal requirements (Livestock Law No. 2005-95, dated October 18, 2005). The study was approved by the Institutional Animal Care and Use committee of the National Agronomic Institute of Tunisia (Certificate No. 09/INAT.DSA.02.02/2023). The research emphasized animal welfare. All experimental procedures were designed to minimize stallions’ pain, distress, and discomfort.

### Study duration and location

The study was conducted from February to May 2023 in northern Tunisia at the Equine Semen Production Center of the National Foundation for the Improvement of the Horse Breed. The center was established in January 2023 and is the first of its kind in Africa. Its mission is to distribute semen from performance-proven stallions via AI and to advance Tunisia’s equine sector.

### Experimental animals

#### Sample animals

Semen from 13 Arabian stallions, seven of Tunisian origin and six of foreign parentage was collected and frozen in the center (total ejaculates = 49). All stallions were clinically healthy and exhibited normal reproductive parameters, as confirmed by prior evaluations at the time of their approval in the center. Their ages ranged from 6 to 20 years (age ≤15 years, n = 5; age >15 years, n = 8).

#### Housing

Stallions were individually housed in 3.5 × 4 × 4 m stalls at the center. Each stall featured a metal door with an upper half composed of bars spaced 0.1 m apart. A feed trough was located 1 m above the ground in each box’s rear corner. The floors were covered with thick straw bedding that was replaced daily.

#### Feeding

Stallions were fed with 5 kg of hay, 5 kg of straw, and 4 kg of concentrate daily. Feed was distributed four times daily: Twice in the morning and twice in the afternoon. Drinking water was supplied automatically, and the water quality was regularly monitored by the center.

### Semen collection

#### Collection method

Semen was collected using a Hannover-type artificial vagina (Minitube, Germany). The artificial vagina was prepared before semen collection by filling it with water at 45°C through a built-in tap. The internal pressure was adjusted to ensure proper penile penetration and to prevent overdilation. A non-spermicidal lubricating gel was applied to the inner surface of the artificial vagina before use. The semen collection bottle was equipped with a sperm filter, and the artificial vagina was kept warm under a heat lamp until collection.

Semen was collected using either a dummy or an estrous mare as a mount. The method of semen collection was predetermined according to each stallion’s known preferences. Stallions that were reluctant to mount the dummy were collected using an estrous mare. An experienced handler supervised the semen collection procedure with minimal stress.

#### Environment

Semen was collected in the center’s designated collection room. The room was well ventilated and maintained at a comfortable temperature. The floor surrounding the dummy mount and semen collection area was covered with a rubber mat to reduce hoof noise and prevent slipping. The collection room was well-lit, and hygiene protocols were followed before each semen collection session.

### Assessment of sexual behavior

#### Behavioral observation protocol

The behavioral responses of the stallion toward the dummy or estrous mare, such as vocalizations, sniffing, kicking, Flehmen, biting, and kicking, were observed and recorded immediately upon entry into the collection room until the completion of the ejaculation phase. The observer had received specific training in the recognition and classification of stallions’ sexual behavior. Behavioral recording was performed manually using a structured observation form.

#### Arousal intensity score

The intensity of sexual arousal toward the dummy or estrous mare was assessed using a subjective scale adapted from Rua *et al*. [12]: (−) disinterest and low activity; (+) low interest and activity; (++) normal interest and activity; and (+++) high interest and activity (Table 1).

**Table 1:** Scale for evaluating sexual arousal intensity in stallions in response to an estrous mare or dummy.

Intensity of sexual arousal	Description
(−) Disinterest and low activity	The stallion shows minimal approach or no physical response to the estrous mare or dummy
(+) Low interest and activity	Occasionally approach the estrous mare or dummy with mild awareness or curiosity
(++) Normal interest and activity levels	Typical sexual behavior with possible attempts to approach the estrous mare or dummy
(+++) High level of interest and activity	Intense sexual arousal with frequent approach attempts, strong activity, and persistent focus on the estrous mare or dummy.

Mounting and copulatory behaviors were recorded during each session of semen collection. The number of mounting attempts on the dummy or estrous mare was recorded. Penile erection was categorized and timed as follows: Level 1 (L1), onset; Level 2 (L2), partial erection; Level 3 (L3), complete erection. Preparation time was defined as the duration from the entry of the stallion into the collection room until the ejaculation. Ejaculation time was calculated by subtracting the preparation time from the total collection time.

### Semen evaluation

#### Fresh semen analysis

Semen volume (mL) was measured by reading the graduation marks on the collection bottle. Semen was diluted with INRA 96® extender (IMV Technologies, France). Sperm motility (%) was evaluated by examining a drop of diluted semen under a phase-contrast microscope (Optech, Optical Technology, Germany) at 10× magnification.

Sperm concentration (millions/mL) was assessed using Thoma cells. A 0.1 mL aliquot of neat semen was diluted 1:40 in a 0.09% formaldehyde solution and evaluated at 40× magnification. Total sperm count (billions) was obtained by multiplying semen volume by sperm concentration.

The same sample was used to assess sperm morphology using the Thoma cells. A total of 200 sperm cells were randomly counted across multiple fields of the counting chamber at 40× magnification.

#### Frozen semen

Semen was frozen following the protocol established by Nationaux [13]. Thawed semen was evaluated after 72 h of storage. Two randomly selected straws per ejaculate were thawed for 30 s in a 35°C –37°C water bath. Straw contents were transferred to a 37°C prewarmed tube containing INRA 96® extender (IMV Technologies) after thawing.

Sperm motility was evaluated using the same procedure as for fresh semen. Sperm viability was assessed using the Vita-Eosine “RAL” KIT (1 × 100 mL Eosin, 1 × 100 mL Nigrosin, Reference 380420-0000, Ral Diagnostics, France). In an Eppendorf tube, 0.5 μL semen was mixed with 0.5 μL eosin and agitated for 30 s. Two drops of nigrosin were added, mixed, and spread on a slide to create a smear, which was examined under phase-contrast microscopy at 40× magnification. Viability was determined by randomly counting 200 spermatozoa; pink-stained cells were classified as dead, whereas unstained cells were considered viable. Sperm morphology was evaluated using the same slide as for viability, following the Samper protocol [14].

The integrity of the plasma membrane was evaluated using the method of Colenbrander *et al*. [15]. A hypotonic solution was prepared by dissolving sodium sulfate (0.735 g) and fructose (1.351 g) in distilled water. The volume was adjusted to reach an osmolarity of 100 mOsm [16]. A 0.4 μL semen sample was diluted in 0.4 μL of the hypotonic solution. The sample was incubated for 20 min in a 37°C water bath. Two drops were mounted between a slide and a coverslip and examined under a light microscope at 40× magnification. Intact spermatozoa displayed characteristic tail swelling and flagellar coiling, identified as hypo-osmotically swollen spermatozoa (HOS+). Sperm with compromised membranes failed to respond to hypoosmotic stress and lacked flagellar coiling. To calculate the HOS+ percentage, 200 spermatozoa were randomly counted per drop (total n = 400) across the slide fields.

### Statistical analysis

Statistical analyses were conducted using SAS software version 9.1.3 (SAS Institute Inc., Cary, NC, USA). A multifactorial analysis of variance was performed using the GLM procedure. The model assessed factors influencing sexual behavior, libido, and semen characteristics in both fresh and frozen samples.

The model was:

Yijkl = μ + Ai + Bj + Ck + Dl + εijkl

Where Yijkl is the dependent variable representing sexual behavior, libido, and semen quality; μ is the overall mean; Ai is the effect of stallion age (≤15 years vs. >15 years); Bj is the effect of stallion origin (Tunisian vs. foreign parentage); Ck is the effect of sexual arousal intensity (−, +, ++, +++); Dl is the effect of collection method (dummy vs. estrous mare); and εijkl is the random error term.

The Student–Newman–Keuls test was used to compare group means for each variable. Statistical significance was set at p < 0.05.

## RESULTS

### Sexual behavioral motivation and libido during semen collection

Behavioral responses of the stallion during semen collection are presented in Tables 2 and 3. Four of the seven recorded behavioral signs varied significantly with the stallion’s origin. Vocalizations, licking, and sniffing of the anonasal and anogenital regions were more frequent in stallions of foreign origin than in those of Tunisian origin (p < 0.05). Furthermore, the frequency of kicking behavior was significantly higher when semen was collected using an estrous mare (p < 0.01). Among all behaviors, only vocalizations were significantly higher in older stallions (p < 0.05). Vocalizations, anogenital sniffing, and Flehmen responses were significantly more frequent in stallions with high (+++) or normal (++) arousal levels (p < 0.05).

**Table 2:** Effect of sexual arousal intensity on sexual behavior signs during semen collection.

Variables	Intensity of sexual arousal				p-value

	(−)	(+)	(++)	(+++)	
Vocalizations	2.4^c^ ± 0.9	4.8^b^ ± 1.2	5.8^a^ ± 1.0	6.4^a^ ± 2.3	0.0095
Licking	0.4 ± 0.01	0.0 ± 0.0	0.5 ± 0.3	0.0 ± 0.0	0.1901
Sniffing of the anonasal region	11.7 ± 5.4	8.2 ± 2.5	8.0 ± 1.6	15.0 ± 8.3	0.6194
Sniffing of the anogenital region	2.0^a^ ± 1.0	0.9^b^ ± 0.3	2.6^a^ ± 1.2	0.0^c^ ± 0.0	0.0392
Flehmen	0.0^b^ ± 0.0	0.7^a^ ± 0.3	1.3^a^ ± 0.5	0.0^b^ ± 0.0	0.0291
Bites	1.4 ± 0.4	1.0 ± 0.3	1.1 ± 0.4	2.4 ± 1.0	0.5498
Kicks	0.0 ± 0.0	0.4 ± 0.1	0.2 ± 0.1	1.7 ± 0.6	0.0001

Each data represents the means ± standard deviation. Different small letters on the rows indicate the difference between the variables according to the Student–Newman–Keuls test (p < 0.05)

**Table 3:** Effect of the stallions’ age and origin and the collection method on sexual behavior signs during semen collection.

Variables	Age		p-value	Origin		p-value	Collection method		p-value
		
	Young	Old		From Tunisian parents	From foreign parents		Dummy	Estrous mare	
Vocalizations	3.6^b^ ± 0.7	6.0^a^ ± 1.0	0.0440	3.9^b^ ± 0.7	6.0^a^ ± 1.1	0.026	4.8 ± 1.0	5.5 ± 1.3	0.2374
Licking	0.5 ± 0.2	0.1 ± 0.06	0.1674	0.0^b^ ± 0.0	0.5^a^ ± 0.3	0.0066	0.3 ± 0.08	0.2 ± 0.02	0.5143
Sniffing of the anonasal region	9.0 ± 1.9	10.3 ± 2.0	0.0801	5.8^b^ ± 1.0	12.0^a^ ± 2.1	0.0061	8.3^b^ ± 2.3	15.6^a^ ± 2.3	0.1748
Sniffing of the anogenital region	3.0 ± 0.9	0.8 ± 0.3	0.2761	0.1^b^ ± 0.09	2.4^a^ ± 0.8	0.0514	1.4 ± 0.4	2.4 ± 0.9	0.4942
Flehmen	0.8 ± 0.4	0.7 ± 0.3	0.8933	0.8 ± 0.4	0.7 ± 0.4	0.2204	0.7 ± 0.3	0.9 ± 0.5	0.8398
Bites	1.0 ± 0.3	1.5 ± 0.4	0.1498	0.6 ± 0.2	1.8 ± 0.4	0.068	1.0 ± 0.3	2.0 ± 0.5	0.1180
Kicks	0.2 ± 0.09	0.5 ± 0.1	0.2070	0.3 ± 0.1	0.4 ± 0.2	0.8344	0.2^b^ ± 0.02	0.9^a^ ± 0.1	0.0007

Each data represents the means ± standard deviation. Different small letters on the rows indicate the difference between the variables according to the Student–Newman–Keuls test (p < 0.05)

The mounting and copulatory behavior results are detailed in Tables 4 and 5. Stallions exhibiting high arousal (+++) mounted the dummy or estrous mare significantly more often (p < 0.01). In contrast, stallions with low arousal (−) had longer partial (L2) and complete (L3) erection durations (p < 0.05), whereas preparation time was longer in highly aroused (+++) stallions (p = 0.05). In addition, some stallions exhibited complete erections before entering the collection room, indicating high arousal toward the dummy or estrous mare.

**Table 4:** Effect of sexual arousal intensity on mounting and copulatory behavior during semen collection.

Variables	Intensity of sexual arousal				p-value

	(−)	(+)	(++)	(+++)	
Number of mounts	1.2^c^ ± 0.1	1.6^c^ ± 0.2	2.1^b^ ± 0.2	3.8^a^ ± 0.9	0.0028
Onset of erection duration (L1) (s)	265.3 ± 102.3	167.6 ± 91.5	49.4 ± 18.6	-	0.0614
Partial erection duration (L2) (s)	272.7^a^ ± 107.3	188.3^b^ ± 90.7	61.4^c^ ± 18.0	-	0.0533
Complete erection duration (L3) (s)	283.7^a^ ± 103.7	208.2^a^ ± 85.9	72.9^b^ ± 18.2	-	0.0475
Preparation duration (s)	261.2^b^ ± 58.4	237.1^b^ ± 60.3	269.0^b^ ± 63.4	410.0^a^ ± 183.4	0.0503
Collection duration (s)	304.1 ± 59.9	274.3 ± 61.7	293.6 ± 62.2	296.2 ± 148.0	0.1726
Ejaculation duration (s)	34.9 ± 3.1	34.4 ± 1.4	28.5 ± 2.6	36.5 ± 6.0	0.2096

Each data represents the means ± standard deviation. Different small letters on the rows indicate the difference between the variables according to the Student–Newman–Keuls test (p < 0.05). L1 = Level 1, L2 = Level 2, L3 = Level 3

**Table 5:** Effect of stallions’ age and origin and collection method on mounting and copulatory behavior during semen collection.

Variables	Age		p-value	Origin		p-value	Collection method		p-value
		
	Young	Old		From Tunisian parents	From foreign parents		Dummy	Estrous mare	
Number of mounts	1.8 ± 0.3	2.5 ± 0.5	0.3092	2.0 ± 0.3	2.4 ± 0.6	0.3634	2.0 ± 0.4	2.6 ± 0.6	0.1195
Onset of erection duration (L1) (s)	72.3 ± 16.3	357.9 ± 109.6	0.5614	376.4 ± 115.1	53.8 ± 12.4	0.0881	421.2^a^ ± 206.9	163.6^b^ ± 60.9	0.0447
Partial erection duration (L2) (s)	91.1 ± 16.0	314.7 ± 98.3	0.8119	391.9^a^ ± 104.2	64.0^b^ ± 13.6	0.0231	429.0^a^ ± 209.7	165.0^b^ ± 53.7	0.0361
Complete erection duration (L3) (s)	310.2 ± 65.6	106.9 ± 10.9	0.5487	134.7 ± 22.2	343.2 ± 73.8	0.0686	442.0^a^ ± 139.5	190.0^b^ ± 35.4	0.0131
Preparation duration (s)	301.6 ± 48.9	288.7 ± 57.9	0.8925	205.7^b^ ± 42.6	395.9^a^ ± 59.2	0.0197	248.6^b^ ± 34.9	478.0^a^ ± 118.7	0.0320
Collection duration (s)	299.1 ± 48.0	325.2 ± 50.0	0.9029	241.0^b^ ± 42.4	403.4^a^ ± 52.0	0.0196	280.2 ± 35.3	460.7 ± 92.1	0.0649
Ejaculation duration (s)	35.0 ± 2.1	29.8 ± 1.7	0.0599	31.1 ± 2.2	34.0 ± 1.7	0.9364	31.7 ± 1.6	36.6 ± 1.5	0.8080

Each data represents the means ± standard deviation. Different small letters on the rows indicate the difference between the variables according to the Student–Newman–Keuls test (p < 0.05). L1 = Level 1, L2 = Level 2, L3 = Level 3

Stallions whose semen was collected using a dummy, exhibited longer durations for onset (L1), partial (L2), and complete (L3) erection stages (p < 0.05). In contrast, stallions collected with an estrous mare showed significantly longer preparation times (p < 0.05).

Stallions of Tunisian origin had longer partial erection durations (L2) (p < 0.05), whereas those of foreign origin required more time for preparation and collection (p < 0.05). Stallion age had no significant effect on mounting or copulatory behavior during semen collection (p > 0.05).

### Semen quality

#### Fresh semen

Tables 6 and 7 present the parameters of fresh semen quality. Sexual arousal intensity significantly influenced semen volume, sperm motility, sperm concentration, and total sperm count (p < 0.05). Stallions with high arousal (+++) produced larger semen volumes. Interestingly, stallions with low arousal (+) had higher motility and concentration, while the highest total sperm count occurred at normal arousal levels (++).

**Table 6:** Effect of the stallion’s sexual arousal intensity on fresh semen characteristics.

Variables	Intensity of sexual arousal				p-value

	(−)	(+)	(++)	(+++)	
Sperm volume (mL)	33.0^b^ ± 10.7	35.0^b^ ± 4.0	44.0^b^ ± 4.7	55.5^a^ ± 2.5	0.0131
Sperm motility (%)	85.0^a^ ± 3.2	70.6^b^ ± 3.6	76.0^b,c^ ± 2.2	67.5^c^ ± 7.5	0.0002
Sperm concentration (millions of spermatozoa)	233.8^a^ ± 64.2	266^a^ ± 60.8	223.7^a^ ± 53.0	172.5^b^ ± 29.5	0.0014
Total number of spermatozoa (in billions)	4.9^b^ ± 0.7	7.6^a,b^ ± 1.0	10.9^a^ ± 2.1	9.8^a,b^ ± 1.2	0.0066
Abnormal sperm count (%)	22.9 ± 5.5	34.9 ± 2.1	36.6 ± 4.0	42.7 ± 15.2	0.5603

Each data represents the means ± standard deviation. Different small letters on the rows indicate the difference between the variables according to the Student–Newman–Keuls test (p < 0.05)

**Table 7:** Variation of fresh semen quality according to stallions’ age, origin, and collection method during semen collection.

Variables	Age		p-value	Origin		p-value	Collection method		p-value
		
	Young	Old		From Tunisian parents	From foreign parents		Dummy	Estrous mare	
Sperm volume (mL)	50.2^a^ ± 4.1	29.6^b^ ± 13.4	0.0001	41.4 ± 2.9	37.4 ± 6.1	0.7648	42.0 ± 3.4	28.30 ± 9.03	0.9748
Sperm motility (%)	72.7 ± 2.7	78.2 ± 2.6	0.9053	69.3^b^ ± 2.5	82.3^a^ ± 1.5	0.0001	73.9^b^ ± 2.1	83.0^a^ ± 3.8	0.0485
Sperm concentration (millions of spermatozoa)	140.0^b^ ± 87.0	360.0^a^ ± 140.0	0.0001	210.0 ± 34.0	250.0 ± 50.0	0.1597	210.1^b^ ± 37.0	360.1^a^ ± 90.1	0.0289
Total number of spermatozoa (in billions)	7.4^b^ ± 14.1	9.0^a^ ± 5.0	0.0154	10.0 ± 1.7	6.8 ± 0.8	0.0893	8.6 ± 1.1	6.0 ± 1.3	0.8217
Abnormal sperm count (%)	39.5^a^ ± 7.0	24.8^b^ ± 3.7	0.0001	35.7^a^ ± 4.1	29.5^b^ ± 7.3	0.0199	33.1 ± 4.3	28.6 ± 11.7	0.5603

Each data represents the means ± standard deviation. Different small letters on the rows indicate the difference between the variables according to the Student–Newman–Keuls test (p < 0.05)

Stallion age significantly affected semen volume, sperm concentration, total sperm count, and the percentage of abnormal sperm (p < 0.05). Young stallions exhibited greater semen volume and higher rates of sperm abnormalities (p < 0.05). Older stallions had higher sperm concentrations (p < 0.05).

Sperm motility was significantly higher in stallions of foreign origin (p < 0.01), whereas sperm abnormalities were more prevalent in those of Tunisian origin (p < 0.01). Semen collected using an estrous mare yielded the highest sperm motility and concentration, outperforming the dummy method (p < 0.05).

#### Frozen semen

Tables 8 and 9 summarize the frozen semen characteristics. Neither the stallion origin nor the collection method had a significant impact on the frozen semen characteristics (p > 0.05).

**Table 8:** Effect of sexual arousal intensity of the stallion on the characteristics of frozen semen.

Variables	Intensity of sexual arousal				p-value

	(−)	(+)	(++)	(+++)	
Sperm motility (%)	50.0^a^ ± 4.2	29.4^b^ ± 4.9	35.0^b^ ± 4.2	32.5^b^ ± 4.3	0.0077
Sperm viability (%)	48.9 ± 4.0	38.4 ± 6.1	49.8 ± 4.8	45.4 ± 3.9	0.4463
Sperm membrane integrity (%)	48.1 ± 1.3	36.5 ± 7.5	41.5 ± 5.7	40.5 ± 0.6	0.4133
Abnormal sperm count (%)	22.1 ± 2.1	25.5 ± 1.4	28.3 ± 2.1	22.5 ± 3.4	0.1837

Each data represents the means ± standard deviation. Different small letters on the rows indicate the difference between the variables according to the Student–Newman–Keuls test (p < 0.05)

**Table 9:** Variation in frozen semen quality according to stallion age, origin, and collection method during semen collection.

Variables	Age		p-value	Origin		p-value	Collection method		p-value
		
	Young	Old		From Tunisian parents	From foreign parents		Dummy	Estrous mare	
Sperm motility (%)	44.3 ± 3.3	39.0 ± 4.6	0.9178	40 ± 2.9	44.4 ± 6.6	0.2456	45.5 ± 3.9	37.2 ± 3.7	0.1633
Sperm viability (%)	48.3 ± 3.9	42.7 ± 4.4	0.3008	46.3 ± 3.3	43.3 ± 6.4	0.8060	46.9 ± 4.0	43.7 ± 0.9	0.7964
Sperm membrane integrity (%)	44.8 ± 4.0	43.7 ± 5.7	0.8920	45.7 ± 4.3	41.2 ± 6.3	0.9964	48.5 ± 5.2	39.3 ± 1.8	0.9553
Abnormal sperm count (%)	22.5 ± 1.4^b^	27.0 ± 1.8^a^	0.01375	27.8 ± 1.4	21.7 ± 1.5	0.6227	26.0 ± 1.9	25.5 ± 1.2	0.2041

Each data represents the means ± standard deviation. Different small letters on the rows indicate the difference between the variables according to the Student–Newman–Keuls test (p < 0.05)

Notably, sperm motility was significantly higher in frozen semen from stallions exhibiting disinterest and low arousal (−) (p < 0.01). In addition, the number of abnormal sperm was greater in old stallions (p < 0.01).

The findings of this study indicated that higher sexual arousal intensity in stallions was associated with enhanced expression of vocalization, anogenital sniffing, and Flehmen response. Stallions exhibiting high sexual arousal also required longer preparation times before ejaculation. Conversely, those with lower sexual arousal produced semen with high sperm motility in both fresh and frozen semen. In addition, sperm motility was significantly improved in stallions of foreign origin and when semen collection was performed using an estrous mare. Notably, the copulatory behavior parameters were longer when semen collection was performed using a dummy. Sperm abnormalities were prevalent in the fresh semen of younger stallions but were reduced in their frozen semen.

## DISCUSSION

### Arousal and behavior

This study demonstrated that sexual arousal intensity influenced vocalizations, Flehmen response, and sniffing behavior in the anogenital region, whether the stallion was presented with a dummy or an estrous mare. A previous study by Stahlbaum and Houpt [17] reported that the Flehmen response is not directly associated with immediate sexual behavior but rather serves to assess the estrous status of mares. Flehmen behavior is typically elicited by the presence of urine from nearby horses [18], and this response is not exclusive to males; mares and even foals can also display it [19]. In addition, certain behavioral expressions, such as vocalizations, are associated with the emotional arousal of the stallion [20]. These findings suggest that environmental conditions may influence the expression of such behaviors. In the present study, sexual arousal intensity appeared to be a direct modulator of behavioral expression, likely shaped by factors associated with the semen collection environment.

### Erection and ejaculation patterns

Arousal intensity influenced the number of mounts, partial erection duration (L2), complete erection time (L3), and preparation duration. One study reported no clear association between erection and heightened arousal, sexual behavior, or aggressiveness in stallions [21]. In contrast, other studies by Yadav *et al*. [3] and Dascanio [10] have shown that mounting and copulatory behavior help trigger libido and are influenced by training and experience levels. Behavioral dysfunction may result from psychological causes or excessive prior breeding use in the absence of physical or hormonal issues [22]. Variations in mounting and copulatory behavior may also be linked to neural activity originating in the cerebral cortex, which affects erection onset and preparation time. A comparative study of natural service versus semen collection suggested that different sensory stimuli activate brain and spinal networks regulating erection, thereby influencing preparation time [23]. Previous findings by Giuliano and Rampin [24] and Melis *et al*. [25] have indicated that certain neurotransmitters, particularly dopamine, play a key role in regulating male sexual arousal and motivation through specific brain and spinal pathways.

Certain behavioral parameters are interrelated, for example, if mounting fails to result in ejaculation, preparation time tends to increase, a trend also observed by Noue *et al*. [23]. Other studies by Waheed *et al*. [9] and Cavinder *et al*. [26] have found that normal breeders exhibit shorter latency to erection and fewer mounts than slow Arab stallions, with sexual behavior parameters correlating with serum Estradiol-17β and cortisol levels. Interestingly, normal sexual behavior has been observed even when serum testosterone levels are low [9].

In this study, stallions showed shorter erection onset durations when semen was collected using an estrous mare. Guillaume *et al*. [18] confirmed that stallions display increased sexual motivation when exposed to an estrous mare. In addition, stallions with low libido or disinterest may suffer from shyness or social inhibition [27, 28].

### Arousal and semen parameters

This study showed that sperm motility and sperm concentration in both fresh and frozen semen were higher when the stallion exhibited low arousal. This may be due to reduced sexual stimulation. High arousal before semen collection may cause prolonged sympathetic activation, which increases oxidative stress and ultimately impairs sperm motility [29].

Most studies have investigated the relationship between sexual arousal and behavioral parameters in stallions; however, none have examined the direct relationship between sexual arousal intensity and semen characteristics. Nevertheless, Houssou *et al*. [30] have reported significant correlations between sexual behavior and semen quality. Shawki *et al*. [31] have shown a negative correlation between sexual behavior and semen parameters, while McDonnell and Love [32] have found no influence of sexual behavior on semen quality after collection. These discrepancies may be attributed to differences in experimental design, stallion populations, and methods for assessing both sexual behavior and semen traits. Such factors highlight the complexity of the interaction between sexual behavior and semen characteristics and emphasize the need for further research to elucidate the mechanisms underlying these relationships.

### Effects of collection method, stallion origin, and age

This study found that sniffing of the anonasal region and kicking were more frequent when semen was collected from an estrous mare. Furthermore, stallions of foreign origin displayed more kicking and sniffing behaviors (anonasal and anogenital), whereas older stallions exhibited more frequent vocalizations. Previous studies by Houssou *et al*. [33], Yadav *et al*. [34], and McDonnell *et al*. [35] have shown that stallion sexual behavior is often influenced by genetic background, environmental exposure, and management practices, which may explain the variation observed in the present study.

In this study, age had no significant effect on copulatory or sexual behavior, consistent with findings for Marwari [34], Brazilian Pony [36], and Mangalarga Marchador stallions [37].

The quality of frozen semen did not differ according to the semen collection method. In contrast, sperm motility and concentration in fresh semen were higher when collection was performed using an estrous mare. Collecting semen from a mare is generally easier, as it requires less training and yields semen that closely mimics natural mating conditions [38]. However, this method can pose safety risks for both stallion and handler [38], as an agitated mare may kick—an event observed in this study.

Stallion age affected the total percentage of abnormal sperm in both fresh and frozen semen. In this study, total abnormal sperm increased in the fresh semen of young stallions, whereas after freezing–thawing, the proportion of abnormal sperm increased in older stallions. Previous studies by Martí *et al*. [39], Carreira *et al*. [40], Abah *et al*. [41], Waheed *et al*. [42], Darr *et al*. [43], and Aurich *et al*. [44] have also reported the impact of age on semen quality in domestic animals and stallions. Sperm resistance to cryopreservation declines with age in stallions [44]. Consequently, as stallions grow older, the likelihood of ejaculates failing to meet quality standards for cryopreservation increases.

### Limitations and methodological considerations

Although this study provided evidence of the effect of sexual arousal intensity on stallion behavior and semen quality, some limitations should be acknowledged. The small number of stallions (13) may reduce the generalizability of the findings and increase the influence of individual variability on the results. The estimation of sexual arousal intensity was based on a behavioral scoring system, which remains partially subjective despite being structured and informed by observable criteria. These factors may help explain some inconsistencies observed in the relationships among arousal intensity, behavior, and semen quality. Future research involving a larger and more diverse sample of stallions, combined with more objective measures of arousal, such as hormonal or physiological indicators, would be valuable in confirming and expanding these findings.

## CONCLUSION

This study provides novel insights into the influence of sexual arousal intensity on copulatory behavior and semen quality in Arabian stallions, highlighting complex interactions with stallion origin, age, and semen collection method. High sexual arousal (+++) was associated with increased vocalizations, anogenital sniffing, and Flehmen responses, as well as longer preparation times before ejaculation. Conversely, stallions exhibiting low arousal (−/+) showed longer complete erection durations and produced semen with superior motility in both fresh and frozen states. Semen collected from an estrous mare yielded higher motility and concentration in fresh samples, while the dummy collection method prolonged erection stages but reduced the risk of handler injury. Stallions of foreign origin exhibited better fresh semen motility but required longer preparation and collection times. Age significantly influenced semen parameters, with young stallions producing larger volumes but more abnormal sperm in fresh samples, whereas older stallions exhibited increased sperm abnormalities post-freezing.

These findings can guide AI centers in refining stallion management protocols. Matching semen collection methods to stallion temperament and arousal profile can improve semen quality while minimizing handling risks. Incorporating behavioral assessments into routine semen evaluation may enhance fertility prediction and breeding program efficiency.

A strength of this study lies in being the first to link graded sexual arousal intensity to both behavioral and semen quality parameters in stallions, while also simultaneously evaluating fresh and frozen semen in relation to behavioral traits and incorporating multiple influencing factors.

However, the small sample size (n = 13) limits generalizability and may amplify individual variability, and sexual arousal intensity was measured through behavioral scoring, which, while structured, retains some subjectivity.

Future research with larger and more diverse stallion populations, incorporating objective physiological indicators of arousal such as hormonal assays for testosterone and cortisol, is warranted to validate and extend these findings. Investigating the neuroendocrine mechanisms underlying the arousal–semen quality relationship could also yield targeted interventions to optimize stallion reproductive performance.

Overall, this study underscores the importance of integrating behavioral assessment into semen collection and AI protocols. By tailoring collection methods and handling practices to individual stallion profiles, AI centers can improve semen quality, enhance breeding outcomes, and ensure animal welfare, while the observed dissociation between behavioral excitement and physiological semen quality opens new avenues for understanding reproductive efficiency in equine breeding programs.

## AUTHORS’ CONTRIBUTIONS

AN: Conceptualization, methodology, data curation, statistical analysis, and writing the original draft. AD: Data curation and statistical analysis. SE: Methodology. BB: Conceptualization. SK: Conceptualization and reviewed the manuscript. All authors have read and approved the final manuscript.
